# Fluorescent garlic-capped Ag nanoparticles as dual sensors for the detection of acetone and acrylamide

**DOI:** 10.1039/d2ra06789g

**Published:** 2022-11-28

**Authors:** Marwa Ahmed El-Naka, A. El-Dissouky, G. Y. Ali, Shaker Ebrahim, Azza Shokry

**Affiliations:** Chemistry Department, Faculty of Science, Alexandria University P.O. Box 426, Ibrahimia 21321 Alexandria Egypt Marwa.ElNaka_PG@alexu.edu.eg; Department of Materials Science, Institute of Graduate Studies and Research, Alexandria University P.O. Box 832 Alexandria Egypt

## Abstract

In order to protect human health from the adverse impacts of acrylamide and acetone, simple analytical processes are required to detect low concentrations of acrylamide and acetone. Dual functional garlic-capped silver nanoparticles (G-Ag NPs) have been used as fluorescent sensors for acrylamide and acetone. This technique depends on the quenching of the photoluminescence (PL) intensity of G-Ag NPs with the interaction of either acrylamide or acetone. This fluorescent probe presented high selectivity toward acrylamide with a wide linear response of 0.01–6 mM with a limit of detection (LOD) of 2.9 μM. Moreover, this probe also acted as a selective and sensitive fluorescent sensor for the detection of acetone in the range of 0.1–17 mM with LOD of 55 μM. The applicability of G-Ag NPs as a proposed sensor for acrylamide was evaluated using a potato chips sample with a recovery percentage of 102.4%. Acetone concentration is also quantified in human urine samples and the recoveries ranged from 98.8 to 101.7%. Repeatability and reproducibility studies for acrylamide and acetone offered relative standard deviation (RSD) of 0.9% and 1.5%, and 0.77% and 1.1%, respectively.

## Abstract 2

G-Ag NPs succeeded to detect and quantify acrylamide and acetone selectively even in presence of other co-existing interfering substances through a fluorescence quenching mechanism.

## Graphical abstract

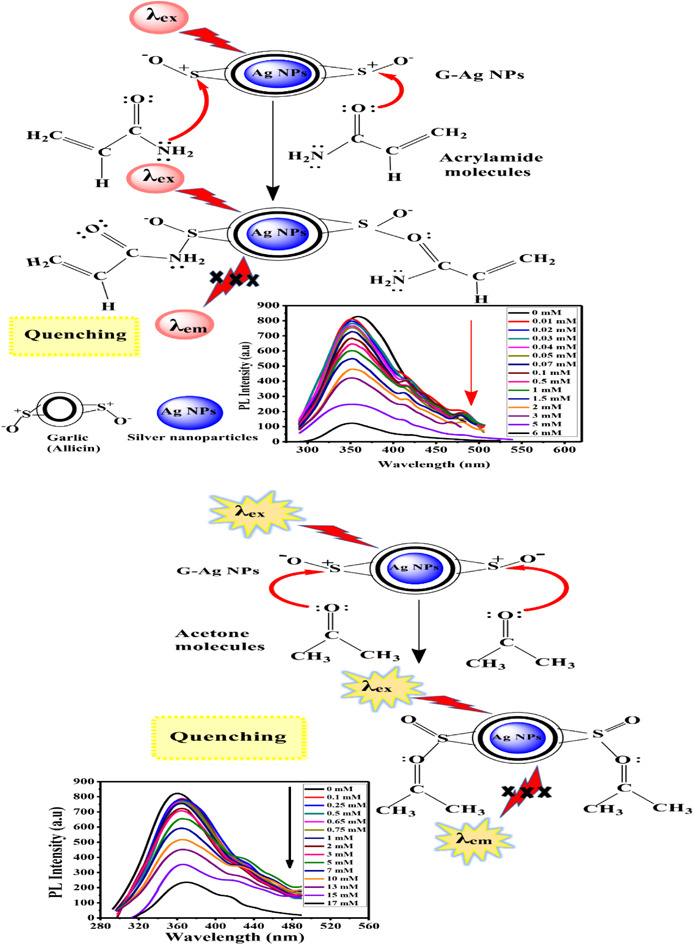

## 1. Introduction

Acrylamide is a white and reactive solid that results from processing and cooking food at a temperature exceeding 120 °C through a reaction called Maillard reaction.^1^ In the Maillard reaction, acrylamide is produced during the cooking of starchy food in which reduced sugars as fructose reacts with asparagine or amino acids during frying, roasting, baking, or, grilling, where temperatures exceed 120 °C.^1,2^ Potato chips, crisp, coffee, and bakery products are the source of one-third of produced acrylamide.^3–11^ Acrylamide is categorized as a group 2A carcinogen by the International Agency for Research on Cancer (IARC).^2,3,12^ According to the European Union (EU), the maximum allowed concentration of acrylamide in food is 0.750 mg kg^−1^ (1.06 × 10^−2^ mM).^12^ The main techniques used for the determination of acrylamide are liquid chromatography-mass spectrometry, ultra-performance liquid chromatography tandem mass spectrometer, gas chromatography, and high-performance liquid chromatography which are all chemicals and time-consuming due to the need for extraction, derivatization, and clean-up steps.^1,13–16^

Acetone is a volatile organic solvent widely used in many industrial applications such as plastics, nail polish remover, rubber, paints, adhesives, and explosives.^17–20^ Exposure to high acetone concentrations is harmful to human health.^21^ World Health Organization determined that prolonged exposure to acetone gas (>2.98 mM) causes dermatitis, eye irritation, skin dryness, throat, cornea or metabolism malfunction, and respiratory and kidney diseases.^17–19,22^ The medical research confirmed that acetone is a biomarker for type-1 diabetes in which the deficiency of insulin in the pancreas induces the body cells to fatty acids breakdown and produce acetone rather than react with glucose.^18,23^ Various techniques have been used for the detection of acetone as acetone vapor detectors such as screen-printed TiO_2_ nanoparticles, silicon doped WO_3_ NPs, MoO–CdO NPs, and ZnO nanoflowers.^18,20,24^ The fluorescence technique to target acrylamide and acetone offers fast response, high sensitivity, excellent selectivity, and good precision with avoiding the disadvantages of the conventional detection methods.^1,11^

Ag NPs are used widely in sensor applications, water treatment, electronics, and antimicrobials due to their distinctive optical, magnetic, and electrical properties. All these properties are attributed to the high surface plasmon resonance of Ag NPs.^25,26^ Garlic (*Allium sativum* L.) is the world's second most widely grown crop, so it is available and cheap.^27^ Garlic aqueous extract contains a variety of phytoconstituents as the negatively charged allicin compound which supports garlic as a capping agent to synthesize nanomaterials. When garlic is used as a capping agent for silver nanoparticles, it offers a sharp PL peak and high PL intensity besides the required stabilization for nanoparticles. Recently, we have reported the synthesis and characterization of highly luminescent garlic-capped silver nanoparticles (G-Ag NPs) for selective and sensitive determination of non-enzymatic cholesterol.^26^

To the best of our knowledge, this is the first time that G-Ag NPs are used as a biosensor for the detection and quantification of acrylamide and acetone. This work aims to use the G-Ag NPs as a dual fluorescent sensor for the detection of dynamic range, the limit of detection (LOD), and sensitivity of acrylamide in the range of 0.01–6 mM, and acetone in the range of 0.1–17 mM. The interference and selectivity of the prepared sensor in the presence of other interfering analytes are investigated. In addition, the suggested mechanisms for the reactions between G-Ag NPs and acrylamide or acetone are proposed and suggested.

## 2. Experimental work

### 2.1. Chemicals and reagents

Acetone (99.0%), urea (99.0%), starch (99.0%), lactose (99.0%), maltose (99.0%) and sodium chloride (99.0%) were purchased from El-Nasr Co., Egypt. Acrylamide and d (+) glucose anhydrous were purchased from BDH Chemicals, England. Acetic acid (99.8%) and sucrose (99.0%) were obtained from Sigma-Aldrich. Calcium carbonate and l-cysteine hydrochloride were obtained from Fishcer Scientific, United States. Uric acid was received from Vitro Scient Co., Egypt. The potato chips samples were obtained from a local supermarket. Human urine samples are provided from a local medical lab.

### 2.2. Preparation of garlic extract

Garlic gloves (10 mg) were washed with DI water, and well-smashed into tiny pieces in a glass mortar followed by adding of 100 mL DI water. This resulting solution was filtered to a white clear solution of garlic extract and concentrated to 92 mL (∼1.6 mM) by heating at 40 °C.^26^

### 2.3. Preparation of G-Ag NPs

G-Ag NPs were synthesized by chemical reduction of silver nitrate using sodium borohydride. Ten millimolar of sodium borohydride (1.2 mL) and 36.8 mL DI water were added to a conical flask and stirred in an ice bath for 10 min. Then, silver nitrate solution (10 mM, 0.4 mL) was dropwise added to the sodium borohydride until the color turned pale yellow indicating the formation of Ag NPs. Garlic extract (1.6 mM, 1 mL) was dropwise added to the resultant solution with continuous stirring for another 10 min.^26^

### 2.4. Characterization techniques

The fluorescence spectra data were collected in the range of 290–500 nm by using a fluorescence spectrophotometer (PerkinElmer LS-55). All required information about the characterization of G-Ag NPs including X-ray diffraction, X-ray photoelectron spectroscopy or high-resolution transmission electron microscopy (were carried out using Bruker-AXS D8 Discover, Thermo Fisher Scientific, USA and JEOL, JEM-2100 LaB6, respectively) and their relevant materials in the present study were reported in our recent research.^26^

### 2.5. Detection of acrylamide and acetone using G-Ag NPs luminescence spectra

The effect of pH on the fluorescence of the prepared G-Ag NPs was investigated in our recent publication and the results revealed that the PL peak of G-Ag NPs in distilled water with a pH of 7.6 has the highest intensity.^26^ For this reason, the detection experiments were conducted at room temperature and pH 7.6. A stock solution of 10 mM of acrylamide was prepared by dissolving acrylamide (0.036 g) in 50 mL of deionized water. Similarly, a stock solution of 20 mM of acetone was prepared by dissolving 0.15 mL of acetone in 100 mL of deionized water. 0.4 mL of G-Ag NPs were mixed with 3.5 mL of various concentrations (0.01–6 mM) of acrylamide, or (0.1–17 mM) of the acetone stock solutions and incubated for 10 min. The samples were utilized for PL measurements at an excitation wavelength (*λ*_ex_) of 270 nm. Acrylamide and acetone were quantified using the maximum photoluminescence (PL) intensities detected at 352 and 365 nm for acrylamide and acetone, respectively.

To examine the selectivity of the G-Ag NPs for acrylamide and acetone, the same experiments were performed with various analytes that behave as interfering agents. For acrylamide, these analytes can exist in potato chips such as glucose, starch, maltose, sucrose, acetic acid, lactose, and sodium chloride with a concentration of 4 mM. The selectivity of G-Ag NPs towards acetone was investigated using 4 mM of some interfering analytes that are existing in human urine such as l-cysteine hydrochloride (cysteine), calcium carbonate, uric acid, and glucose.

Each measurement was repeated three times. The error bars were calculated from standard deviations. Finally, we apply G-Ag NPs for acrylamide and acetone sensing in potato chips and real human urine samples, respectively. For the optimization of analytical parameters, the calibration curve slope was used depending on eqn (1).^28^

1 (*F*_0_ − *F*)/*F*_0_ = *aC* + *b*

where *F*_0_ and *F* refer to the PL intensity of G-Ag NPs in the absence and presence of acrylamide or acetone, *a* and *b* represent the calibration curve slope and intercept, respectively. *C* refers to the concentration of acrylamide or acetone. The quenching efficiency (QE) was calculated using the following equation:^28,29^

2 QE = (*F*_0_ − *F*)/*F*_0_

The limit of detection (LOD) of acrylamide or acetone was determined using eqn (3).3
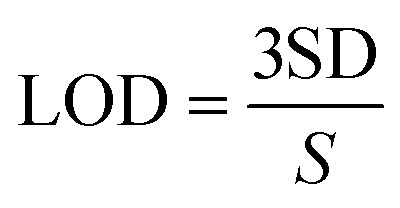
where SD and *S* are standard deviation and slope, respectively.^30^

The recovery (%) was also calculated using eqn (4) to detect the accuracy of the current sensor in real samples.^31,32^4
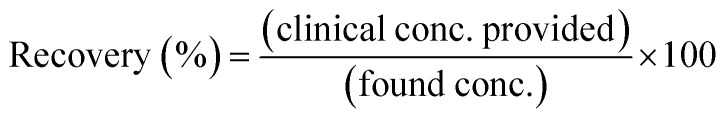


### 2.6. Potato chips sample preparation

Four grams of commercial potato chips sample were weighed and ground with a mortar. The sample was defatted twice by adding 10 mL hexane, mixed with a vortex for 5 min, and decanted by filter paper. The filtrate was dried to get rid of hexane. To extract the acrylamide sample, 0.1 mL of deionized water and 20 mL of acetone were mixed with the defatted dried sample. The sample was put in an ultrasonic bath for 20 min at 40 °C, then filtered. Moreover, evaporation of 10 mL of this filtrate occurred using a vacuum oven at 40 °C followed by adding 2 mL of deionized water and then shaken well. The potato chips sample was then ready and kept in the refrigerator for further use.^15,33^

### 2.7. Determination of acrylamide in the chips sample with HPLC technique

The HPLC of model Agilent 1260 and the column type of C18 Zorbax were used. 14 mM of acrylamide solution was prepared as a stock solution by dissolving 0.1 g of acrylamide in 100 mL of deionized water. Then, the working standard solutions of acrylamide (7.0 mM, 1.4 mM, 0.1 mM, 0.01 mM) were prepared by the proper dilution of the stock solution. The column was injected with a 20 μL injection loop. Also, the temperature of the column remains constant at 40 °C. The flow rate was 0.15 mL min^−1^. The analysis was done at 202 nm and the retention time was 8 min in the case of acrylamide. A calibration curve was deduced from the measurements in which the unknown concentration of the chips sample was determined.^33^

### 2.8. Determination of acetone in the urine samples in the clinical laboratory

To detect acetone levels in a local clinical laboratory, Mission Expert Urinalysis Strips was used. Urine samples of diabetic patients were provided, collected in a tube and measured without pretreatment by immersing the test reagent strip for 2 min. According to the reference range provided with the strips, (+++) 15 mM refers to highly increased concentration of acetone, (++) 10 mM refers to increased and (+) 5 mM refers to slightly increased.^34^

## 3. Results and discussion

### 3.1. Characterization

The morphological properties, structure, and crystallinity of G-Ag NPs were characterized, presented, and discussed in our previous published work.^26^ X-ray photoelectron spectroscopy of G-Ag NPs confirmed the presence of metallic Ag and O, C, and S elements of garlic. Fourier transform infrared presented the distinctive functional groups of G-Ag NPs. The energy-dispersive X-ray showed the elemental analysis of G-Ag NPs: a strong sharp peak at 3 keV of Ag NPs, two moderate peaks at 0.51 keV and 0.25 keV of O-atom and C-atom, and a small peak at 2.3 keV of S-atom. X-ray diffraction pattern of G-Ag NPs confirmed the presence of metallic Ag and their crystalline nature. The high-resolution transmission electron microscopy images and the electron diffraction pattern confirmed the presence of polycrystalline and uniformly distributed spherical shape of G-Ag NPs particles with a diameter size ranging from 4.52 to 12.8 nm, and a *d*-spacing of 0.22 nm. Also, the stability of G-Ag NPs was confirmed after measuring the intensity of their absorption peaks over 1.0 year using the UV-visible spectrophotometer. The zeta potential value of G-Ag NPs was −22 mV which confirms the structural stability of G-Ag NPs. To further confirm the stability of fluorescent G-Ag NPs, the PL intensity of G-Ag NPs was evaluated for 20 weeks. G-Ag NPs showed high stability in PL with only 27.4% decay after 20 weeks.^26^

### 3.2. Fluorescent quantitative analysis of acrylamide

**Fig. 1:**
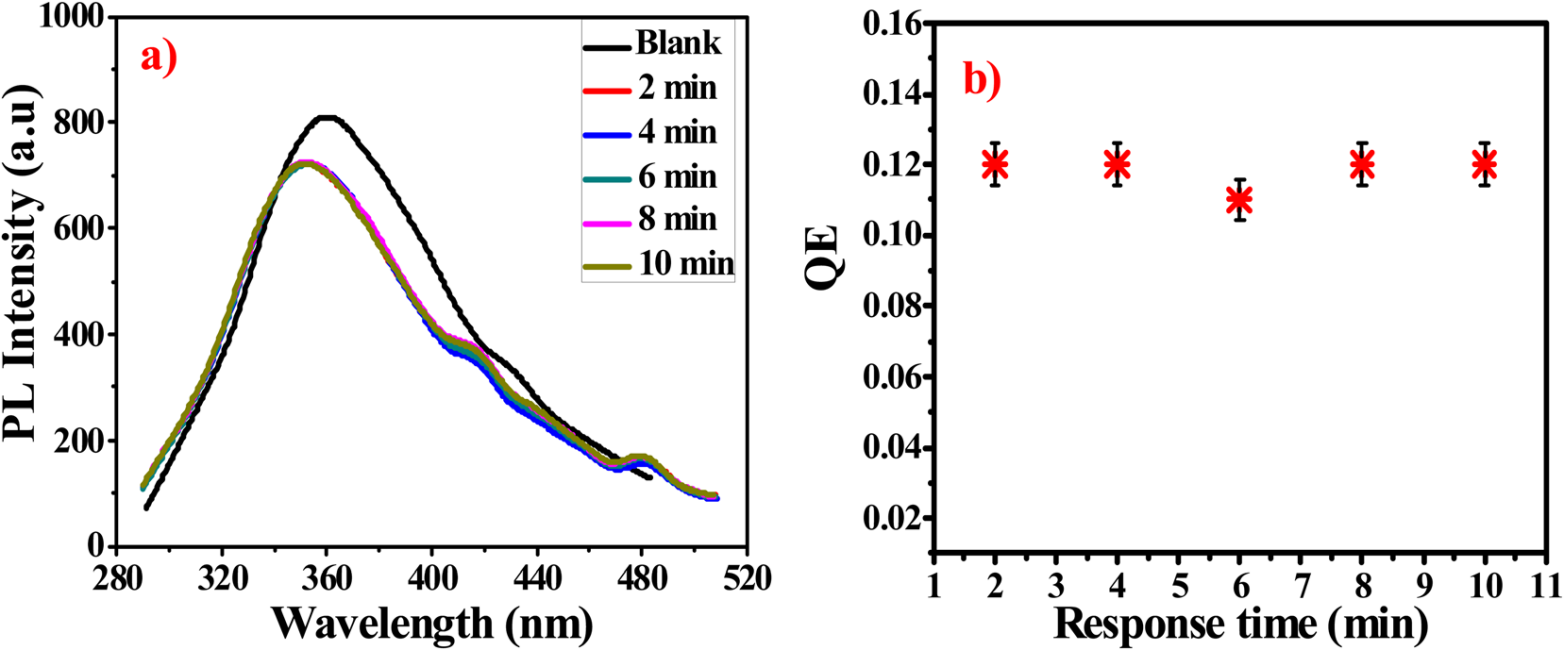
(a) PL intensity of G-Ag NPs in the presence of 0.07 mM acrylamide at different times, (b) QE against response time at *λ*_ex_ = 270 nm.

To obtain optimum detection performance for acrylamide, the effect of incubation time between acrylamide and prepared sensor is studied and presented in Fig. 1a and b. The PL intensity is quenched very fast. The equilibrium state is attained within nearly 2 min. The previous results indicate that the prepared probe is very reactive toward acrylamide which means that G-Ag NPs are stable in addition to their rapid detection.^35^

**Fig. 2:**
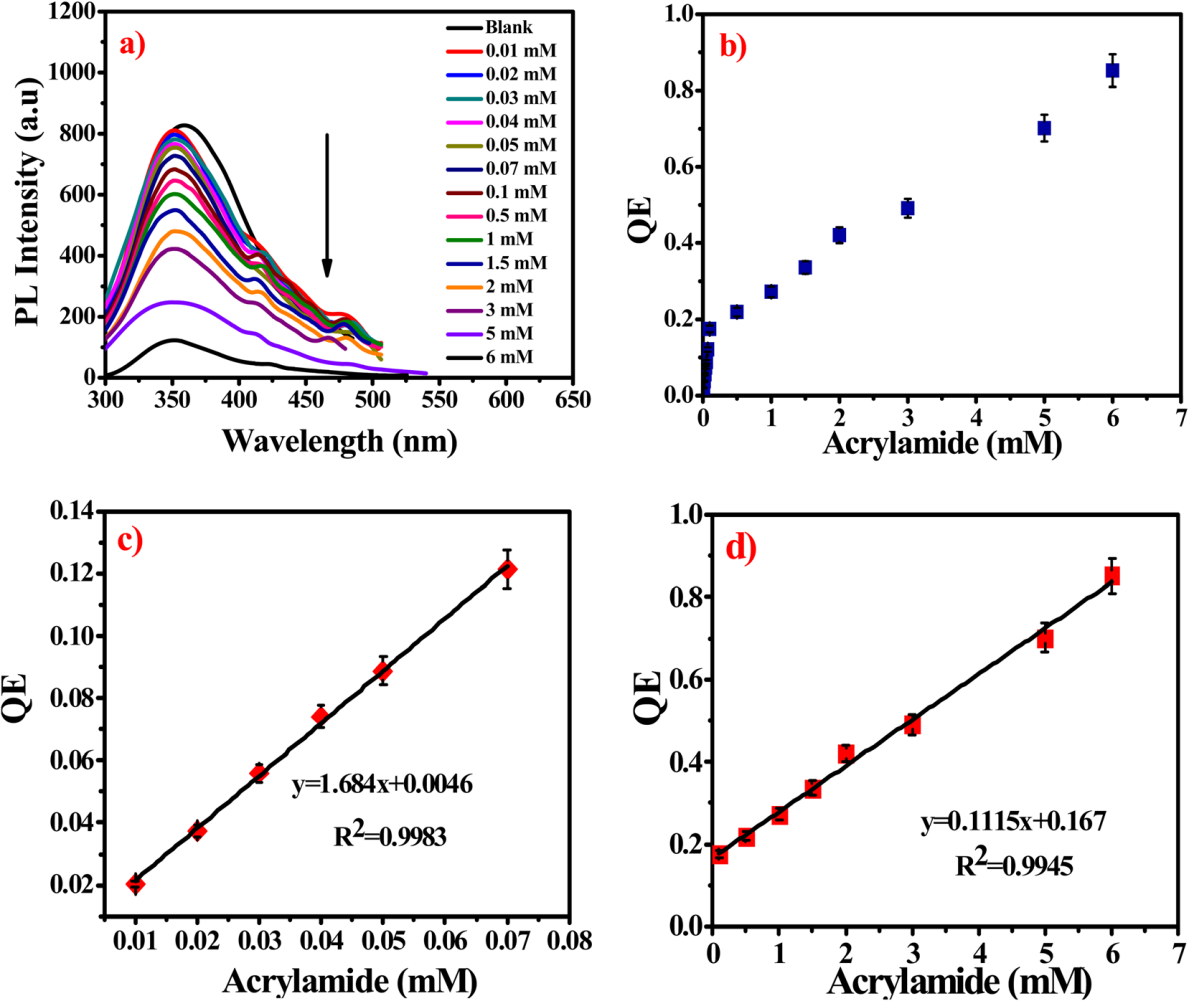
(a) Fluorescence spectra of G-Ag NPs with different concentrations of acrylamide, and QE *versus* acrylamide concentration from (b) 0.01 to 6 mM, (c) 0.01 to 0.07 mM and (d) 0.1 to 6 mM at *λ*_ex_ = 270 nm.

To study the relationship between PL intensity of G-Ag NPs and the acrylamide concentration, 10 mM of acrylamide aqueous solution was prepared, and different concentrations of acrylamide solutions were prepared with a fixed total volume of 3.5 mL. Then, 0.4 mL of the prepared fluorescent G-Ag NPs was added to each acrylamide concentration sample and incubated for 2 min. Ag NPs exhibit a strong and sharp peak near 352 nm and small bands between 500 and 700 nm with different concentrations of acrylamide as recorded and presented in Fig. 2a. Ag NPs have size smaller than the wavelength of light, will respond as a dipole in an optical field. The dominant proposed mechanism to explain the luminescence of Ag NPs is the photoelectron interaction at the surface energy states and absorbs light at its plasmon resonance frequency. There are different molecular forces such as dispersion, induction and tropism forces between Ag NPs. Hybrid energy band of NPs converts from primary energy band to form new superamolecular interface energy bands with a lower energy at longer wavelength. The plasmon energy bands shift depends on the coupling strength and the energy gap between primary ones. This hybridization assumption explains the variations of the surface energy bands of Ag NPs at 480 nm. PL intensity of the blank G-Ag NPs in the absence of acrylamide at 352 nm is gradually quenched more obviously with increasing acrylamide concentration from 0.01 to 6 mM. Moreover, two shoulder peaks at 415 nm and 480 nm appear with a blue shift in the position of the PL peak of about 8 nm due to the complex formation between acrylamide and G-Ag NPs.^28^ At high concentration of acrylamide above 5 mM these peaks are vanished due to the formation of superamolecular interface energy bands.^36^

Fig. 2b–d illustrates two linear relationships between the quenching efficiency (QE) obtained at *λ*_ex_ of 270 nm and concentration of acrylamide in the ranges of 0.01–0.07 mM (*R*^2^ = 0.9983) and 0.1–6 mM (*R*^2^ = 0.9945). This demonstrates that the quantitative determination of acrylamide using a G-Ag NPs probe with different sensitivities (the slope of the calibration lines) of 1.684 and 0.1115 mM^−1^ can be performed according to the range of detection. From the calibration line in Fig. 2c, the LOD was calculated to be 0.0029 mM (2.9 μM) which is lower than the maximum allowed amount of acrylamide in food detected by the EU.^12^ There are different proposed PL quenching mechanism including non-radiative surface defects that occur due to the surface reaction with the quencher. Furthermore, the transfer of charge from the radiative material to the quenching material may be another main mechanism.^37^

#### Mechanism of fluorescent detection of acrylamide

**Fig. 3:**
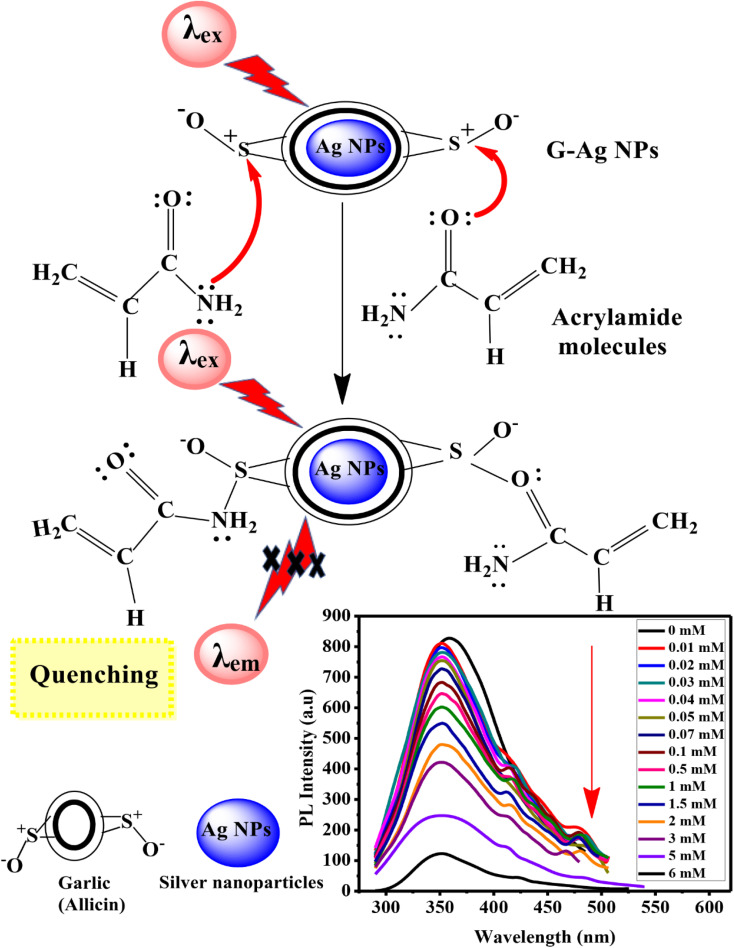
Schematic illustration of the suggested mechanism of the interaction between acrylamide and G-Ag NPs.

The fluorescence detection mechanism of G-Ag NPs is illustrated in Fig. 3. The detection mechanism depends on the binding interaction through the lone pair of –NH_2_ and C=O groups of the acrylamide with the sulfur cation of allicin of the garlic extract of G-Ag NPs. These interactions lead to the formation of a non-phosphorescent complex that decreases the intensity of the emission peak at 352 nm.^1,2,25^

#### Repeatability and reproducibility studies

**Fig. 4:**
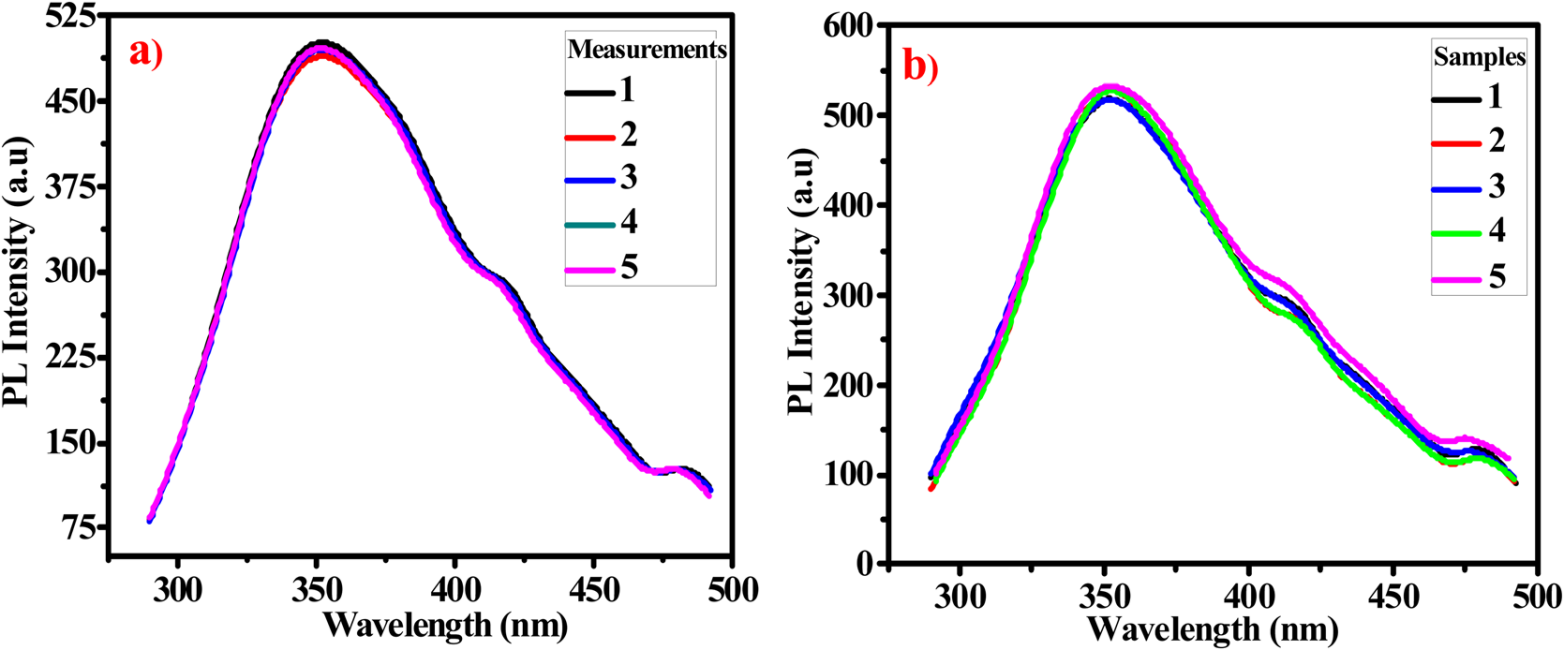
(a) The repeatability of G-Ag NPs for 5 independent PL measurements of a sample containing 2 mM acrylamide, and (b) reproducibility G-Ag NPs for PL measurements of 5 different samples containing 1.75 mM acrylamide.

The fabricated probe repeatability is evaluated by measuring the PL intensity of a same sample of G-Ag NPs mixed with 2 mM acrylamide aqueous solution over five consecutive days (Fig. 4a). The relative standard deviation (RSD) is determined to be 0.9%. To determine the reproducibility of G-Ag NPs, the PL intensities for five different samples of G-Ag NPs added to 1.75 mM acrylamide prepared separately are measured as shown in Fig. 4b. The RSD is calculated to be 1.5%. These results showed that the proposed G-Ag NPs probe is highly reproducible and can be applicable for repeatable experiments.^38,39^

#### Selectivity and interferences of G-Ag NPs for acrylamide

**Fig. 5:**
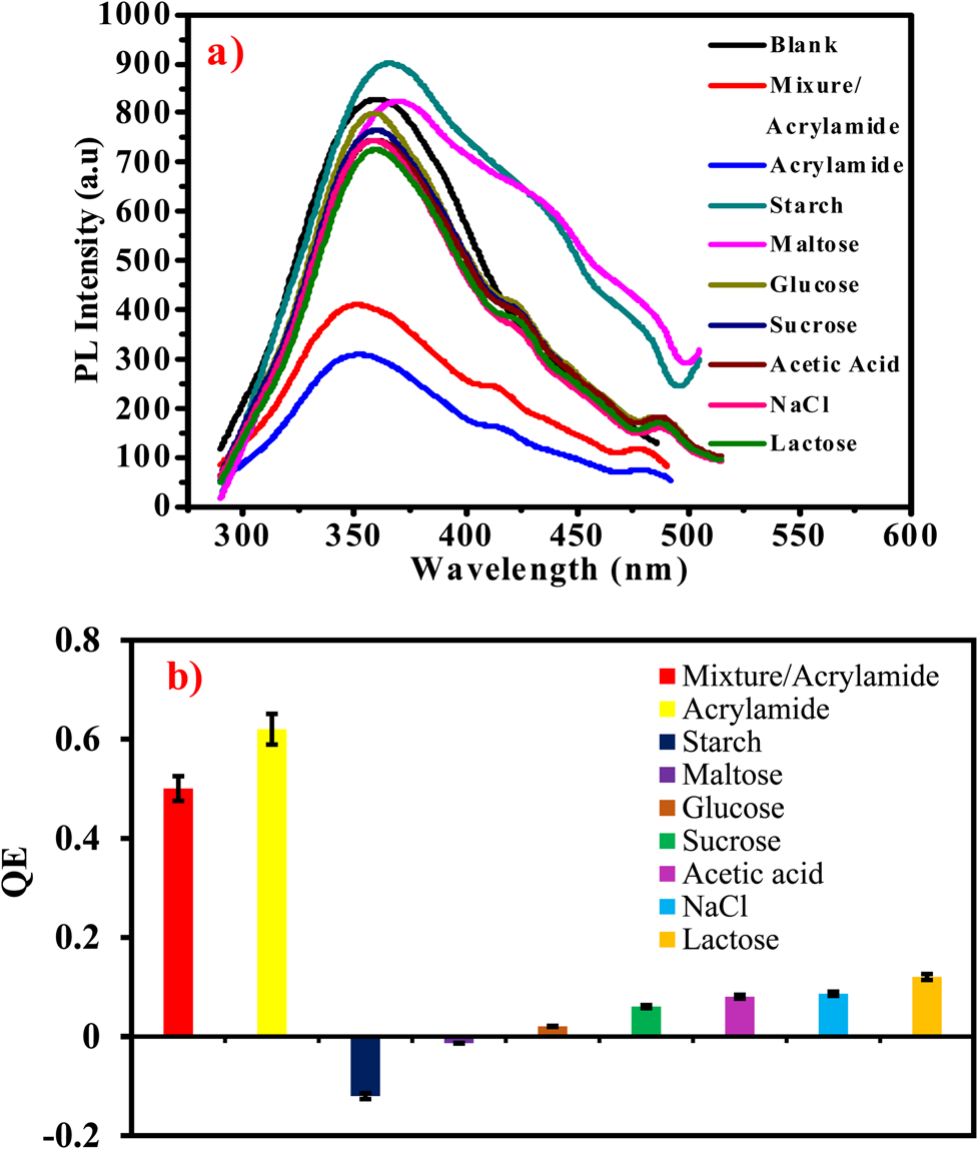
(a) PL intensity of G-Ag NPs in presence of 4 mM of different interfering analytes and (b) QE of G-Ag NPs *versus* 4 mM of different interfering substances, *λ*_ex_ = 270 nm.

The prepared probe selectivity is examined in the presence of potential interfering analytes. These analytes already co-exist with acrylamide in the food samples as glucose, starch, maltose, sucrose, acetic acid, lactose, and sodium chloride. Fig. 5a and b shows the PL spectra and QE of G-Ag NPs after adding 4 mM of several analytes. It is observed that glucose, acetic acid, NaCl, sucrose, and lactose have a small and incomparable quenching influence on the PL intensity with acrylamide. On the other hand, starch and maltose have PL enhancement responses. To investigate the selectivity of the prepared sensor in the presence of interferences, an aqueous solution of 4 mM acrylamide is added into aqueous solutions containing G-Ag NPs and a mixture of 4 mM of each analyte, and the changes in the fluorescence intensity are monitored. The small change in the relative QE of the G-Ag NPs from 0.6 to 0.5 appear as a result of interfering analytes compared to the sample containing only acrylamide in deionized water. From these results, G-Ag NPs as a sensor can be applied in real food samples due to their high selectivity for acrylamide.^40^

#### Detection of acrylamide in potato chips sample

**Fig. 6:**
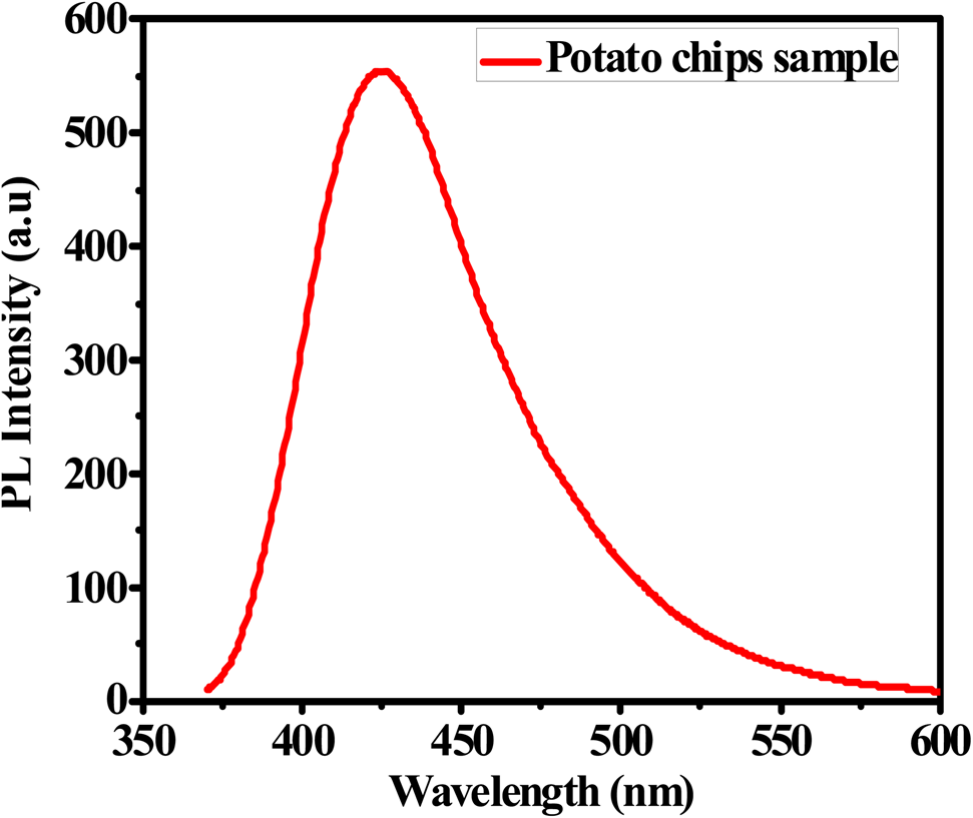
PL spectrum of potato chips sample mixed with G-Ag NPs at *λ*_ex_ = 270 nm.

The practical application of the G-Ag NPs biosensor is examined to quantify acrylamide content in a commercial sample of potato chips. The amount of acrylamide in this sample is measured using the HPLC technique and found to be 1.43 mM. The PL spectrum of the potato chips sample mixed with G-Ag NPs is measured as shown in Fig. 6. The acrylamide concentration is determined using the calibration curve in Fig. 2d to be 1.46 mM. The recovery (%) is calculated and found to be 102.4%. This experiment is conducted 4 times, and the RSD is calculated and found to be 1.75%. The previous results manifest that the prepared G-Ag NPs can be applied as an accurate and efficient probe of acrylamide concentrations in food samples.^31,32^

### 3.3. Fluorescent quantitative analysis of acetone

**Fig. 7:**
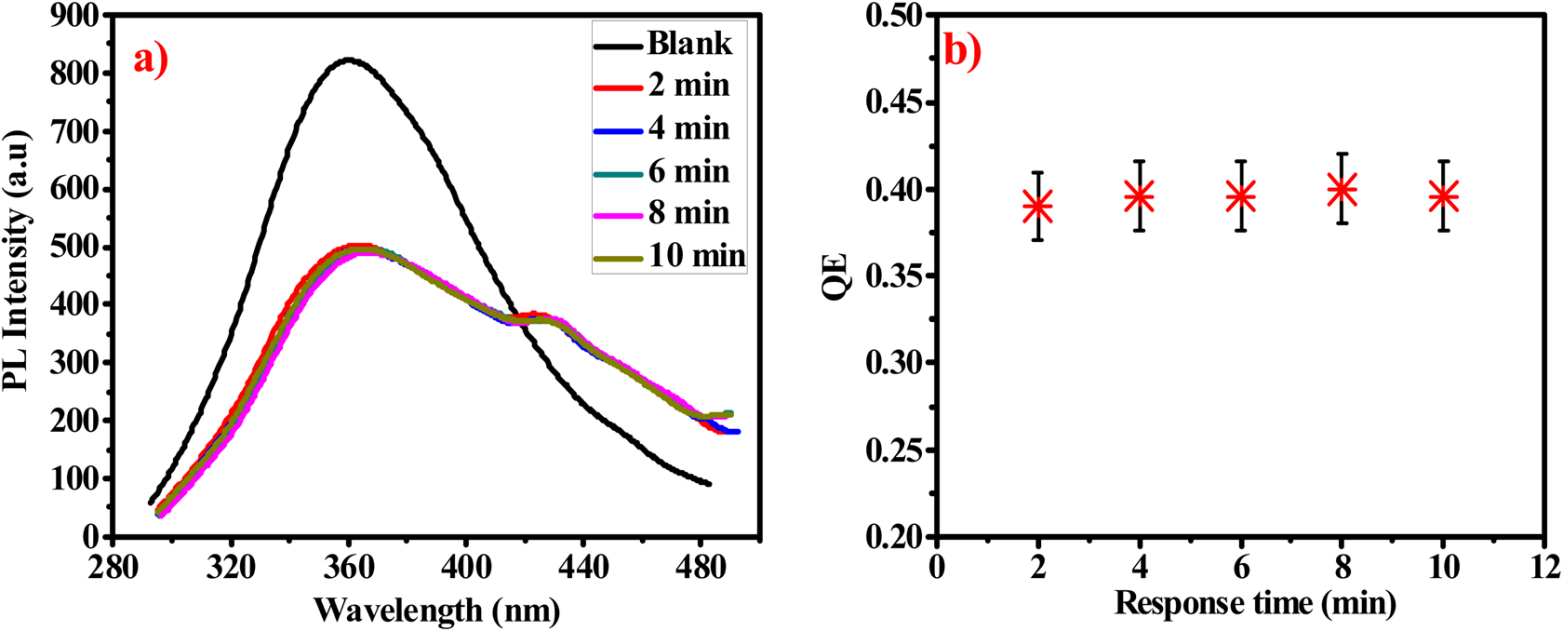
(a) PL spectra of G-Ag NPs in the presence of 10 mM acetone at different times, and (b) QE against response time at *λ*_ex_ = 270.

The excellent luminescence properties of G-Ag NPs have inspired us to further use this probe to detect acetone in aqueous solutions. The response time of acetone quenching is firstly investigated. Fig. 7 shows the change in PL intensity before and after 10 mM of acetone is added. The results indicate that the maximum photoluminescence QE can be achieved within 2 min of acetone addition and remains constant afterward.^35^ The fast-quenching property allows the use of G-Ag NPs for acetone sensing.

**Fig. 8:**
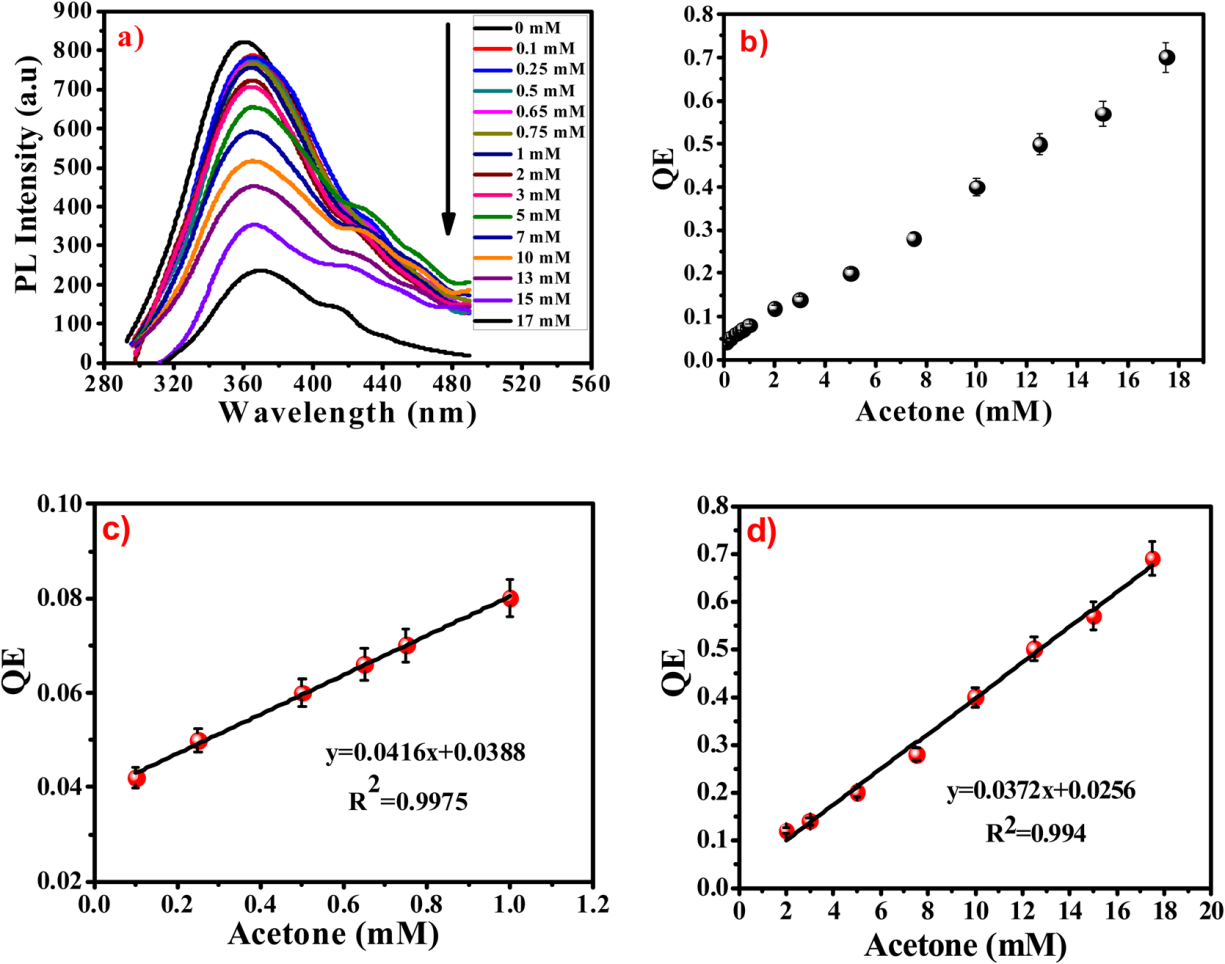
(a) Fluorescence spectra of G-Ag NPs with different concentrations of acetone, and QE *versus* acetone concentration from (b) 0.1 to 17.5 mM, (c) 0.1 to 1 mM, and (d) 2 to 17.5 mM at *λ*_ex_ = 270 nm.

The experimental results of the acetone sensing at different concentrations using the PL quenching effect of prepared sensor are shown in Fig. 8a. The intensity of the PL at the emission peak of 365 nm is largely dependent on the acetone concentration. Notably, increasing of acetone concentration from 0.1 to 17 mM causes the gradual decline in the PL intensity of the G-Ag NPs blank accompanied by 5 nm red shift in the peak position. In addition, a shoulder peak at 431 nm is observed. This occurs as a result of the binding of acetone with the surface functional groups of the G-Ag NPs and complex formation.^41^ It is found that there is good two linear relationships between the concentration of acetone at 0.1–1 mM (*R*^2^ = 0.9975) and 1–17.5 mM (*R*^2^ = 0.9940), and the fluorescence intensity (Fig. 8b–d). This demonstrates that the quantitative detection of acetone using G-Ag NPs probe can be carried out with different sensitivities of 0.0416 and 0.0372 mM according to the detection range. The LOD is calculated from the calibration line (Fig. 8c) to be 0.0548 mM (∼55 μM).

#### Mechanism of fluorescent detection of acetone

**Fig. 9:**
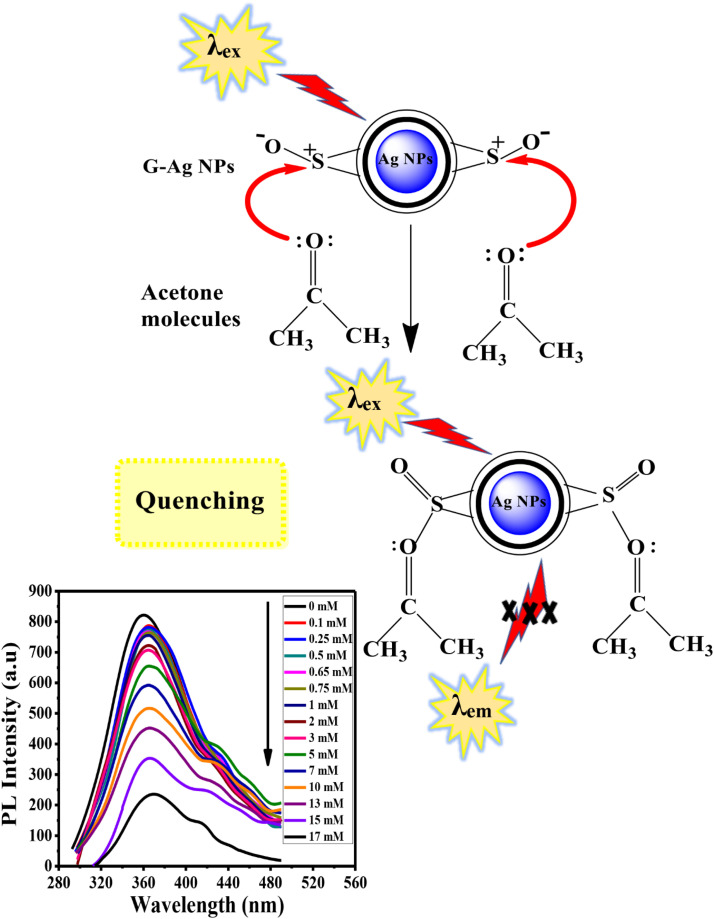
Schematic illustration of the proposed mechanism of interaction between acetone and G-Ag NPs.

Acetone is an excellent electron donor molecule causing irreversible loss of a definite proportion of fluorescence during the interaction of acetone with G-Ag NPs. The quenching mechanism depends on the donation of the lone pair of electrons of the C=O group of acetone to the sulfur cation of allicin of G-Ag NPs.^19^ The prepared G-Ag NPs can be considered a potent optical sensor for acetone (ketone) detection. Fig. 9 represents the suggested quenching mechanism.

#### Repeatability and reproducibility studies

**Fig. 10:**
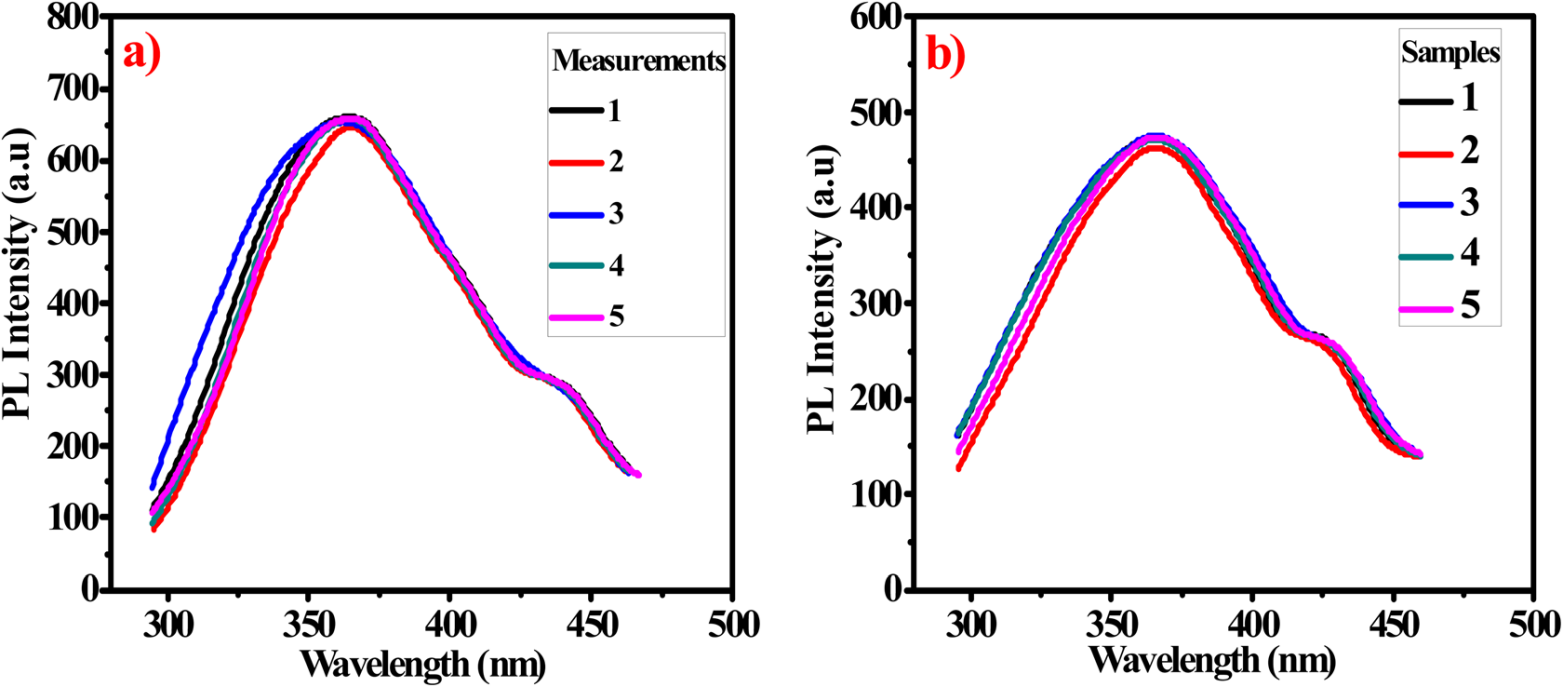
(a) The repeatability of G-Ag NPs for 5 independent PL measurements of a sample containing 4.75 mM acetone, and (b) reproducibility G-Ag NPs for PL measurements of 5 different samples containing 10.75 mM acetone.

Fig. 10a shows good repeatability for five replicate measurements for the PL of the same sample of G-Ag NPs added to 4.75 mM acetone over five days. The obtained RSD is 0.77%. To evaluate the reproducibility, measurements of the PL intensities of five different G-Ag NPs mixed with 10.75 mM acetone are depicted in Fig. 10b. The RSD is found to be 1.1% indicating that this G-Ag NPs probe is highly reproducible and applicable.^39^

#### Selectivity and interferences of G-Ag NPs for acetone

**Fig. 11:**
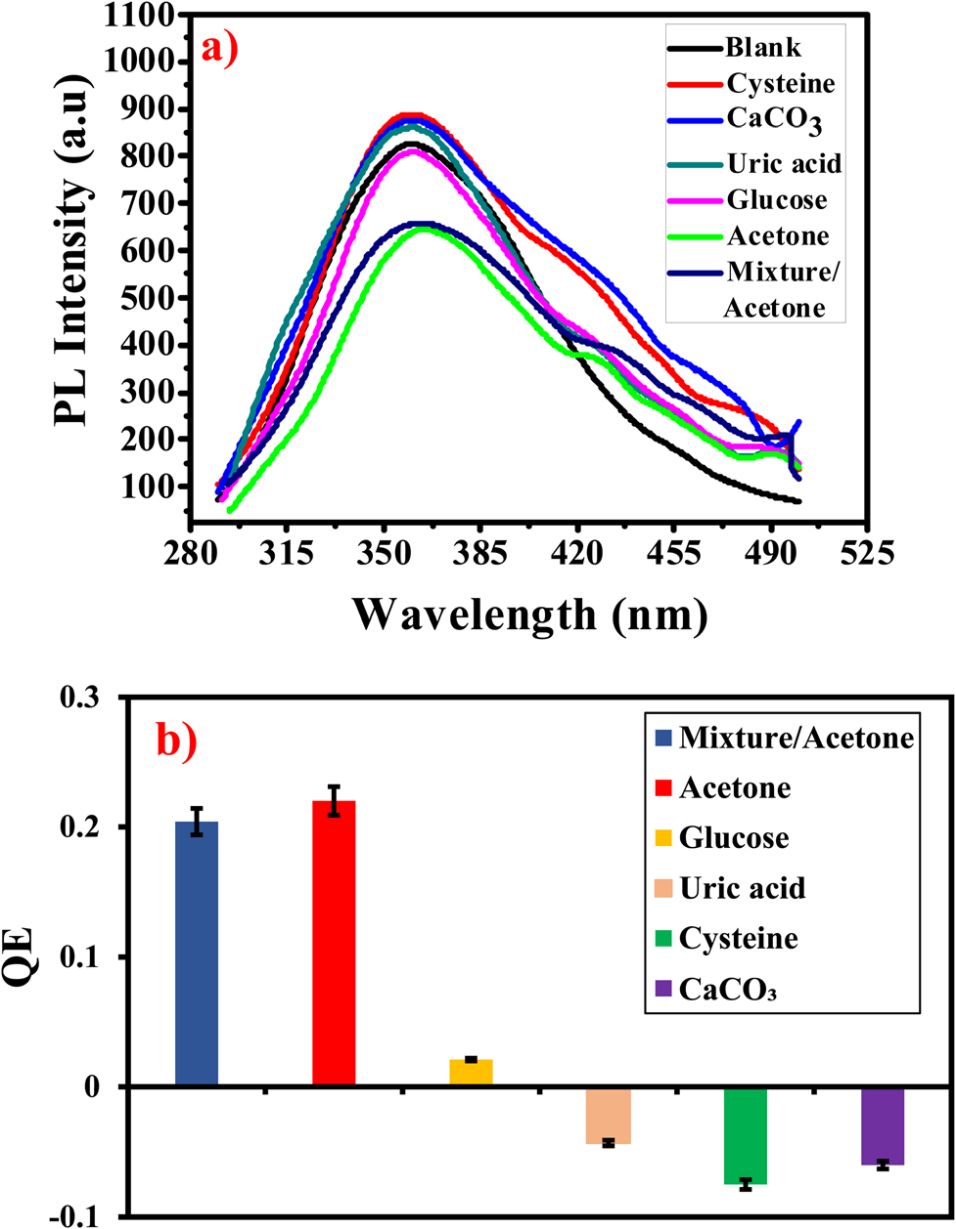
(a) PL intensity of G-Ag NPs in presence of 5 mM of several interfering analytes and (b) QE of G-Ag NPs *versus* 5 mM of different interfering substances at *λ*_ex_ = 270 nm.

The prepared sensor selectivity is evaluated in the presence of l-cysteine hydrochloride (cysteine), calcium carbonate, uric acid, glucose, and their mixture which already co-exist with acetone in the human urine. Fig. 11a and b shows the PL spectra of the prepared probe mixed with 5 mM of several substances. It is observed that glucose has a negligible quenching effect (about 2%) on G-Ag NPs photoluminescence. In contrast cysteine, CaCO_3,_ and uric acid have a mild enhancement influence on the PL intensity. While acetone has a strong PL quenching effect. It is concluded that the prepared sensor is highly selective to acetone so can be applicable in real urine samples.^40,42^

#### Detection of acetone in human urine samples

**Fig. 12:**
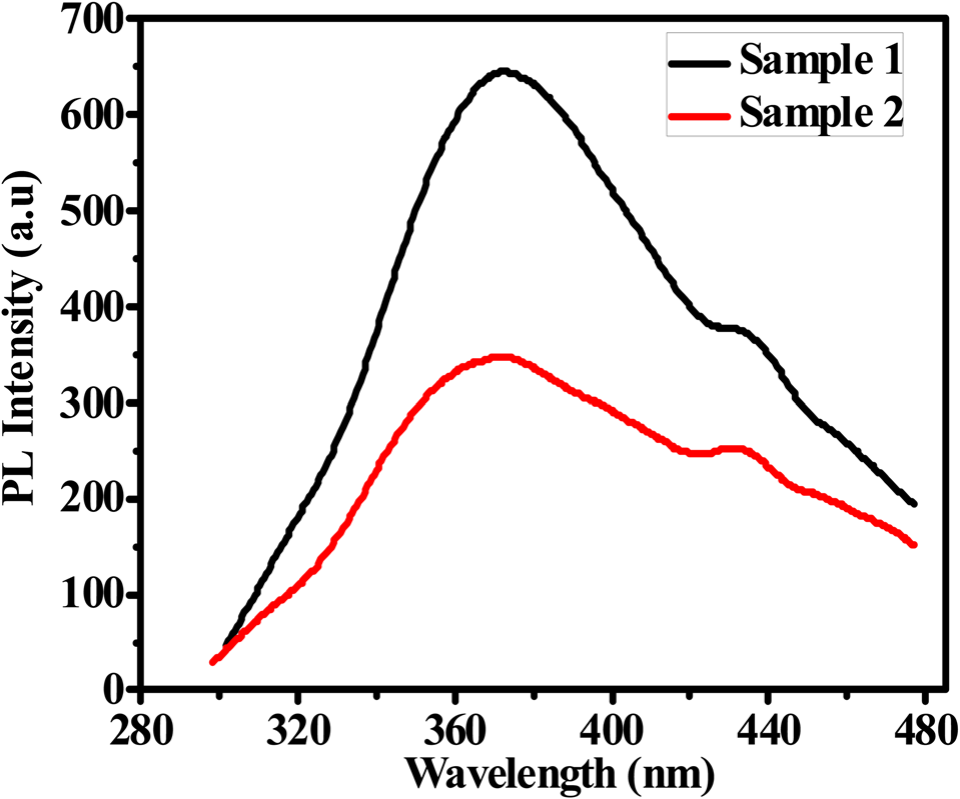
PL spectra of human urine samples at *λ*_ex_ = 270 nm.

The developed G-Ag NPs fluorescent probe is employed for the detection of acetone in two specimens of human urine samples. These samples and their clinical ketone level data (determined by Mission Expert Urinalysis Strips) are provided by a local medical laboratory. Also, the fluorescence of each urine sample is measured four times (Fig. 12). The concentration of acetone is determined through the calibration curve shown in Fig. 8c. Table 1 illustrates the determined acetone concentration using the proposed sensor and the clinical data provided by the laboratory for each sample. The recovery percentages are calculated in the range of 98.8–101.7%. Also, the RSD is detected in the range of 0.7–1.2%. The previous results have proved that the prepared probe can be used as an accurate and efficient sensor for determination of acetone concentrations in human urine.

**Table 1:** The recovered and measured acetone in human urine samples using the developed G-Ag NPs sensor

Sample	Given clinical data (mM)	Found (mM)	Recovery (%)	RSD (%)
1	5	5.09	101.7	1.2
2	15	14.82	98.8	0.7

## 4. Conclusion

G-Ag NPs were applied as a dual functional sensor to quantify acetone and acrylamide concentrations based on the quenching of PL intensity. This probe was used to determine acrylamide and acetone concentration with high selectively and accuracy. Detection limit of 2.9 μM for acrylamide was achieved, which was lower than the maximum allowed amount of acrylamide in food according to the EU and 55 μM for acetone. The findings of this work revealed that the G-Ag NPs probe was selective, sensitive, stable, repeatable, and reproducible. The applicability of the developed G-Ag NPs as acrylamide and acetone sensor have been confirmed by the results obtained from a commercial potato chip and two human urine samples.

## Author contributions

Marwa A. El-Naka: conceptualization, data curation, formal analysis, investigation, methodology, writing – original draft. A. El-Dissouky: conceptualization – supervision – review & editing. G. Y. Ali: supervision – review & editing. Shaker Ebrahim: conceptualization, supervision, data curation, formal analysis, review & editing. Azza Shokry: conceptualization, supervision, data curation, formal analysis, review & editing.

## Conflicts of interest

The authors declare that they have no known competing financial interests or personal relationships that could have appeared to influence the work reported in this paper.

## Supplementary Material
